# Modulation of Adjuvant Arthritis by Cellular and Humoral Immunity to Hsp65

**DOI:** 10.3389/fimmu.2016.00203

**Published:** 2016-06-13

**Authors:** Eugene Y. Kim, Malarvizhi Durai, Younus Mia, Hong R. Kim, Kamal D. Moudgil

**Affiliations:** ^1^Department of Microbiology and Immunology, University of Maryland School of Medicine, Baltimore, MD, USA; ^2^Department of Pharmaceutical Sciences, Washington State University College of Pharmacy, Spokane, WA, USA; ^3^Department of Pharmacy Services, University of Maryland Medical Center, Baltimore, MD, USA; ^4^Chong Kun Dang Pharmaceutical Institute, Yongin-si, Korea; ^5^Department of Medicine, Division of Rheumatology, University of Maryland School of Medicine, Baltimore, MD, USA; ^6^Baltimore VA Medical Center, Baltimore, MD, USA

**Keywords:** heat shock proteins, arthritis, T cells, antibodies, autoimmunity, Hsp60, Hsp65

## Abstract

Heat shock proteins (Hsps) are highly conserved, and their expression is upregulated in cells by heat and other stressful stimuli. These proteins play a vital role in preserving the structural and functional integrity of cells under stress. Despite the ubiquitous expression of Hsps in an individual, the immune system is not fully tolerant to them. In fact, Hsps are highly immunogenic in nature, and immune response to these proteins is observed in various inflammatory and autoimmune diseases. Studies on the immunopathogenesis of autoimmune arthritis in the rat adjuvant arthritis (AA) model of human rheumatoid arthritis (RA) as well as observations in patients with RA and juvenile idiopathic arthritis (JIA) have unraveled immunoregulatory attributes of self-Hsp65-directed immunity. Notable features of Hsp65 immunity in AA include protection rather than disease induction following immunization of Lewis rats with self (rat)-Hsp65; the diversification of T cell response to mycobacterial Hsp65 during the course of AA and its association with spontaneous induction of response to self-Hsp65; the cross-reactive T cells recognizing foreign and self homologs of Hsp65 and their role in disease suppression in rats; the suppressive effect of antibodies to Hsp65 in AA; and the use of Hsp65, its peptides, or altered peptide ligands in controlling autoimmune pathology. The results of studies in the AA model have relevance to RA and JIA. We believe that these insights into Hsp65 immunity would not only advance our understanding of the disease process in RA/JIA, but also lead to the development of novel therapeutic approaches for autoimmune arthritis.

Adjuvant arthritis (AA) is a well-established model of human rheumatoid arthritis (RA) (1–3), and it has extensively been used both for studying arthritis pathogenesis and for testing new anti-arthritic drugs. AA can be induced in inbred Lewis (RT.1^l^) rats by immunization with heat-killed *Mycobacterium tuberculosis* H37Ra (Mtb) (1). AA manifests as a polyarthritis after about 8–10 days of Mtb immunization. The disease rapidly progresses to reach a peak phase by about days 14–16, followed by spontaneous regression of inflammation over the next 10–12 days. Following recovery, AA generally does not relapse or exhibit flares that are typically seen in many RA patients. In this regard, AA differs from RA. Mycobacterial heat shock protein 65 (Bhsp65) is one of the disease-related target antigens in AA (1–3). Studies on immune responses to Hsp65 have offered critical insights into both induction and regulation of autoimmune arthritis (3–9). Arthritic rats raise T cell response to Bhsp65, and the T cells directed against the epitope region 180–188 of Bhsp65 (B180) can adoptively transfer AA to naive rats (2, 10). However, the T cells reactive against certain other epitopes of Bhsp65 are disease-regulating in nature. In our studies in AA summarized here, we have addressed several important questions. For example, are Lewis rats tolerant to self-Hsp65?; how does activation of self-Hsp65-reactive T cells affect the development of arthritis?; how does immune response to foreign Hsp65 evolve during the course of AA?; what is the significance of the T cell repertoire against foreign Hsp65 that is cross-reactive with self-Hsp65?; what role do antibodies to Hsp65 play in AA?; and does Hsp65 treatment influence arthritis induced by a non-antigenic compound? These aspects of cellular and humoral immunity to Hsp65 are elaborated below, based on results of studies by others and us. (Mammalian Hsp60 is a mitochondrial protein of 61 kDa, whereas Hsp65 is a mycobacterial protein of 65 kDa. Hsp60 is used as a synonym for Hsp65, and *vice versa*, therefore, for simplicity, we have used Hsp60 and Hsp65 interchangeably in this article.)

## The Role of Self (rat)-Hsp65 in Arthritis Pathogenesis: Immune Reactivity to Self-Hsp65 is Protective Rather than Pathogenic

**Figure 1:**
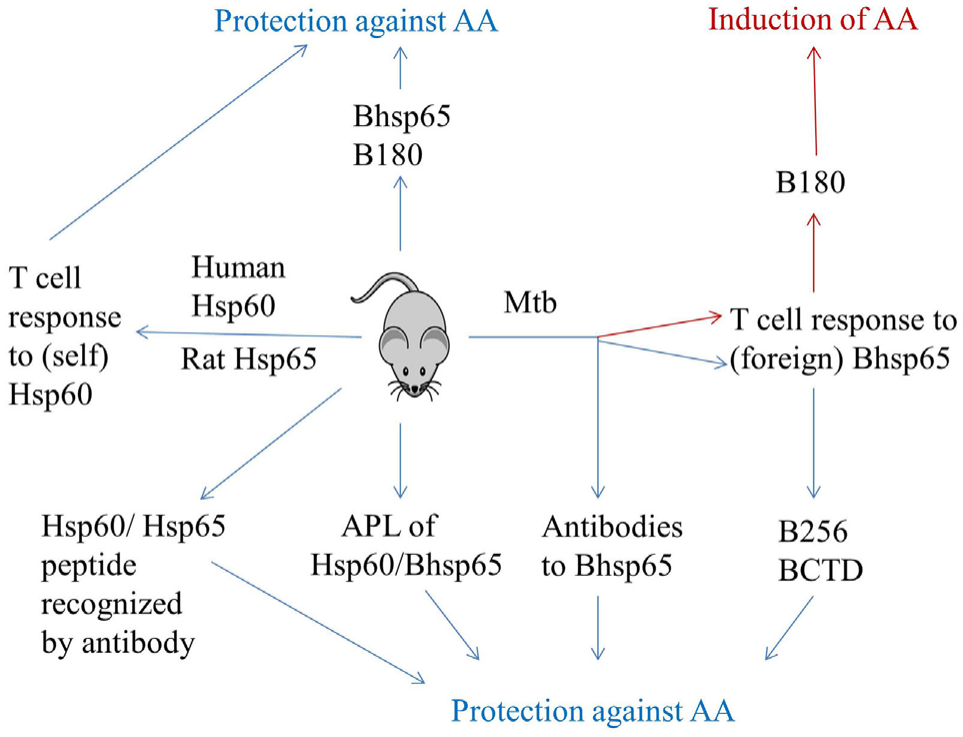
**Induction and regulation of adjuvant arthritis by immune response to Hsp65**. AA can be induced in Lewis rats by subcutaneous immunization with heat-killed *M. tuberculosis* H37Ra (Mtb). Mycobacterial hsp65 (Bhsp5) is one of the targets of immune response in arthritic rats. The T cells against epitope 180–188 of Bhsp65 (B180) are pathogenic, whereas those against other epitopes of Bhsp65 [e.g., 256–270 (B256) and Bhsp65 C-terminal determinants (BCTD)] are regulatory in AA. Tolerization of rats with soluble Bhsp65 or B180 renders these rats relatively resistant to induction of AA by subsequent Mtb injection. Arthritic rats also raise antibody response to Bhsp65, and antibodies have been shown to be protective against AA. Intriguingly, immunization of Lewis rats with self-Hsp65 [e.g., human Hsp60 or rat Hsp65 (Rhsp65)] is protective rather than pathogenic. Similarly, induction of antibody response by a peptide of self-Hsp60 also affords protection against AA. Finally, challenge with altered peptide ligands (APLs) of Bhsp65 and self-Hsp60 can induce a disease-protective response in arthritic rats. Thus, immunity to Hsp65 has dual attributes: pathogenic as well as protective. (This figure summarizes information from multiple studies described in the text.)

As autoimmune reactivity is generally driven by an endogenous self-antigen, we directed our study on AA pathogenesis to rat Hsp65 (Rhsp65) (9, 11). Ours was the first study to examine the state of immune tolerance to Rhsp65 in the Lewis rat. Surprisingly, Lewis rats were not fully tolerant to Rhsp65 (11). The rats challenged with Rhsp65 raised a potent response to this self-antigen, and the pre-treatment of naive rats with Rhsp65 afforded protection against subsequent induction of AA by Mtb injection (11). These results showed that immune response to systemically administered self-Hsp65 is protective against arthritis instead of being pathogenic (Figure 1). Our studies further revealed that Rhsp65 C-terminal determinants (RCTD) are displayed as dominant epitopes following the processing and presentation of native Rhsp65 (11). Furthermore, peptides comprising RCTD, when administered into Lewis rats in a synthetic adjuvant, also induced a T cell response that is protective against AA (11). Thus, anti-self-Hsp65 immune response is protective against AA.

## Immune Response to Human Hsp60 Induces Protection Against Arthritis in Rats

Studies using DNA vaccination approach have revealed the disease-regulating activity of human Hsp60 in AA (Figure 1) (12). Human Hsp60 was more effective than Bhsp65 in suppressing AA. This protective effect was associated with increased interferon-γ (IFN-γ) and transforming growth factor-β (TGF-β) response to Hsp60, but reduced IFN-γ response to B180 (12). Subsequent experiments identified Hu3 as a regulatory epitope within human Hsp60 (13). Immunization with human Hu3, but not its mycobacterial homolog, was protective against AA, and the protective response involved both Th1 and Th2/3 cells (13). A cross-reactive T cell response to human Hsp60 was also invoked in the protective effect of DNA vaccination using Hsp70/Hsp90 (14). Another set of studies have highlighted the role of human Hsp60 in activation of B cells (15), T cells (16), CD4^+^CD25^+^ regulatory T cells (Treg) (17), and maturation of dendritic cells (18), in part *via* Toll-like receptor 4 (TLR4) (B cells)/TLR2 (T cells and Treg) signaling. Furthermore, acting in a different manner, Hsp60 expressed on activated T cells can render them targets of regulatory T cells (19). In an entirely different approach based on CD8^+^ regulatory T cells instead of CD4^+^ regulatory T cells, it was shown that Hsp60(p216) peptide-specific CD8^+^ T cells restricted to a class I-like MHC molecule Qa-1 in mice (HLA-E in humans) effectively suppressed CIA (20). Thus, besides self-Hsp60 epitopes recognized by CD4^+^ T cells, others recognized by CD8^+^ T cells may also contribute to regulation of autoimmune arthritis. Thus, Hsp60 can modulate both innate and adaptive immune response through multiple modes of action, and as for rat Hsp65, immunity to human Hsp60 also induced protection against AA. Further insights into the T cell repertoire against self-Hsp60 and maintenance of tolerance would help advance understanding of the pathogenesis of arthritis and its control (21).

## Immunomodulatory Immune Reactivity to Human Hsp60 in JIA and RA Patients

In a study on patients with JIA, the response to Hsp60 of peripheral blood mononuclear cells (PBMC) from oligoarticular JIA was compared with that of polyarticular JIA. The former group revealed increased expression of CD30 on T cells along with increased interleukin-10 (IL-10)/IFN-γ ratio, whereas the latter group showed no expression of CD30 and low IL-10/IFN-γ ratio (22). Furthermore, remission from acute oligoarticular JIA was attributed to Hsp60-reactive T cells. In another study, it was reported that serum human Hsp60 predicts remission in JIA (23). In a study on PBMC of RA patients, it was observed that pan-DR-binding human Hsp60 peptides induced 5- to 10-fold higher IL-10/tumor necrosis factor-α (TNF-α) ratio than that by microbial peptides, indicating immunomodulatory activity of self-Hsp60 peptides (24). Thus, self (human)-Hsp60 not only induced protection against AA but also contributed to remission in JIA.

## Foreign–Self-Hsp65 Cross-Reactivity and Spreading Control of Arthritis in Rats

Lewis rats treated with Bhsp65 prior to disease induction are protected from subsequent AA (25). Unlike Mtb-immunized rats, which raise T cell response predominantly to epitope B180, Bhsp65-treated rats raised response to multiple additional epitopes besides B180 (25, 26). Epitope 256–270 was among the nine epitopes identified upon Bhsp65 pre-treatment. The T cells reactive against mycobacterial 256–270 were found to be both cross-reactive with self counterpart (Rhsp65 256–270) as well as disease-regulating in nature (26). The T cells against this peptide could be restimulated by stressed antigen-presenting cells (APCs), indicating that the self-ligand recognized was processed and presented from endogenous Hsp60. Furthermore, the treatment of rats either with this peptide or with the T cells primed with it resulted in protection against AA (Figure 1). This cross-reactivity between homologous foreign and self-Hsp60 has been implicated in a spreading regulatory control during the course of arthritis (6). Self-Hsp60-reactive T cells can contribute to immune regulation in different ways. For example, such cells may be anergized, and these anergic cells mediate suppressive effects. Alternatively, self-Hsp60 epitopes may serve as altered peptide ligands (APLs) and induce a regulatory response, while their corresponding microbial Hsp60 ligands may act as potent agonists (6). Thus, T cells activated by Bhsp65, but cross-reactive with self-Hsp65, displayed anti-arthritic activity against AA.

## The Diversification of T Cell Response to Bhsp65 During the Course of AA

Rats with AA undergo spontaneous regression of inflammation. However, the immunological basis of this phenomenon remains to be fully explained. Our study revealed the diversification of T cell response to Bhsp65 during the course of AA (3). Arthritic rats in the late phase of AA displayed new T cell reactivity against BCTD compared to rats in early phase of the disease. Furthermore, synthetic peptides containing the sequences of BCTD, when administered into Lewis rats with a synthetic adjuvant, induced protection against subsequent AA (3). Similarly, the adoptive transfer of BCTD-primed T cells reduced the severity of AA in recipient arthritic rats (27). Thus, deliberate priming and expansion of the T cells against BCTD led to protection against AA (Figure 1). This was the first report on regulatory epitope spreading in autoimmunity because an earlier report on epitope spreading in the EAE model described it as pathogenic (3, 28). Thus, our study expanded the scope of the impact of epitope spreading in autoimmunity and presented a framework to explain natural regression of inflammation in AA.

## Defining the Mechanism of Spontaneous Regression of AA

We observed that BCTD represent cryptic epitopes of Bhsp65, meaning that these epitopes are potentially immunogenic as peptides, but are not efficiently revealed upon processing of the native foreign antigen (Bhsp65) injected into Lewis rats (27). On the contrary, RCTD represent the dominant epitopes of Rhsp65, implying that these epitopes are efficiently displayed following the processing and presentation of the native self-antigen administered into Lewis rats (11). Yet, BCTD can prime T cells that are cross-reactive with the self counterpart. We therefore explained regulatory epitope spreading by suggesting as well as providing evidence that inflammation accompanying acute arthritis upregulates Rhsp65 expression; its RCTD are then displayed to induce a T cell response; and these T cells can then suppress the progression of arthritis, as evident from adoptive transfer experiments using Rhsp65- or RCTD-primed T cells (3, 11, 27, 28). We further suggested that inflammation also upregulates the display of cryptic BCTD, which then can induce T cells that cooperate with RCTD-primed T cells in suppressing pathogenic T cell response, leading to natural recovery from acute AA (Figure 1). Responses to BCTD are also involved in environmental modulation of AA (29).

## Unexpected Cross-Reactivity Between the Pathogenic and the Protective T Cell Epitopes of Homologous Hsp65 in AA

**Figure 2:**
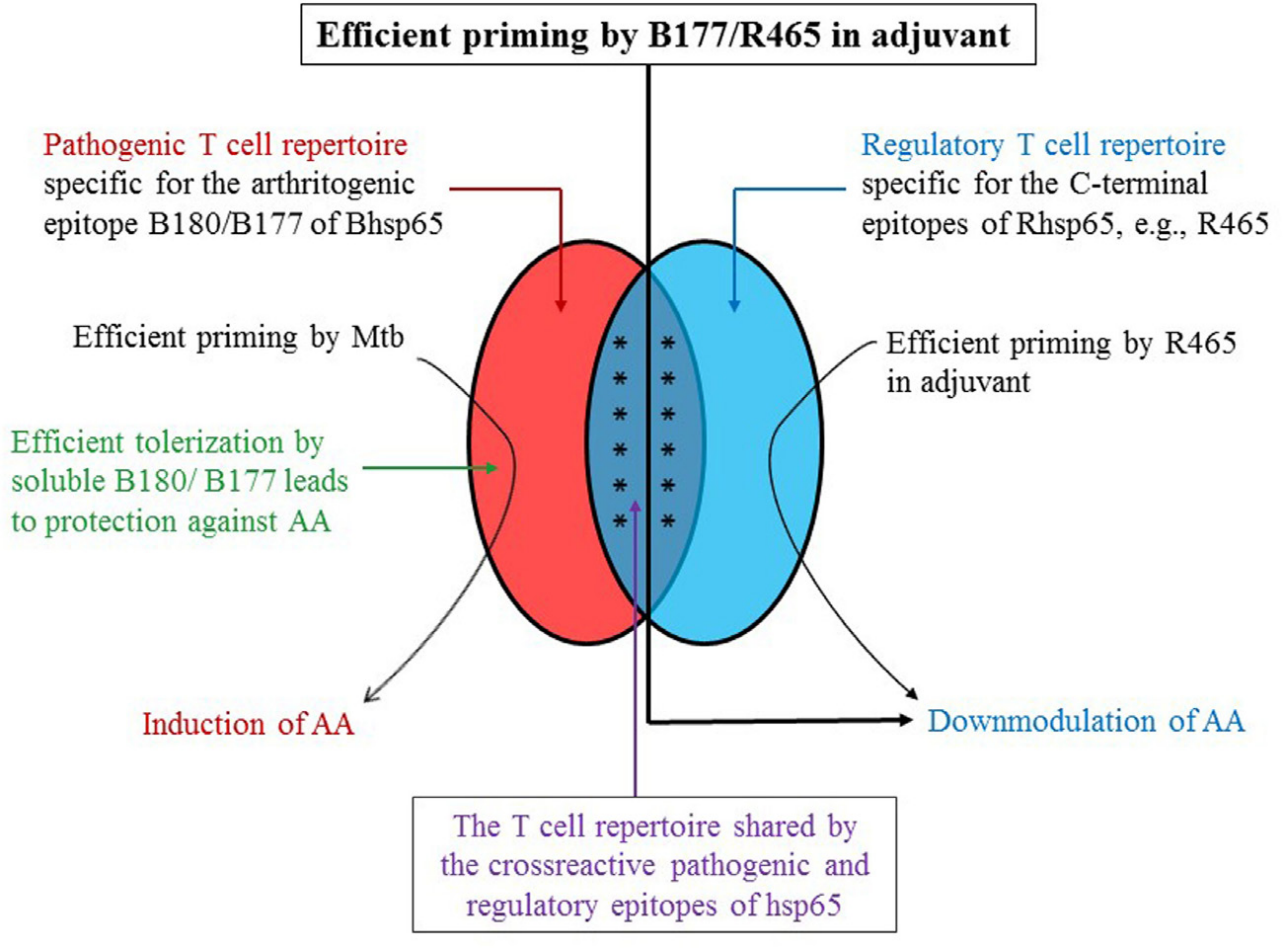
**A schematic representation of the role of cross-reactive T cells directed against B180/B177 and R465 in regulation of AA**. The T cells potentially reactive against B180/B177 of Bhsp65 (pathogenic epitope) and R465 of Rhsp65 (regulatory epitope) comprised a cross-reactive T cell subset besides the corresponding epitope-specific subset. The priming and expansion of these cross-reactive T cells by immunization of Lewis rats either with peptide (B180/B177 or with R465) in adjuvant would induce protection against subsequent AA (“vaccination”). However, injection of Mtb, which contains Bhsp65, instead activates predominantly the B180/B177-specific pathogenic repertoire, resulting in the induction of AA. Furthermore, this pathogenic repertoire may be inactivated, or immune-deviated, by soluble antigen (B180/B177) administered i.p. or nasally, leading to protection against AA. This schematic figure is based on our results published elsewhere (3, 11, 27, 30).

Peptide 177–191 of Bhsp65 (B177) contains the arthritogenic epitope B180 (2), whereas peptide 465–479 of Rhsp65 (R465) represents a regulatory epitope for AA (30). Immunization of rats with B177 in incomplete Freund’s adjuvant or CpG s.c. prior to Mtb injection showed marked suppression of arthritis instead of disease induction (30). We reasoned that the T cell subset activated by B177 injected in IFA/CpG is distinct from that activated by Mtb challenge; the latter subset mediates induction of arthritis, and that there might be some connection between B177 and one of the regulatory C-terminal determinants of Hsp65, including R465 described above. In fact, surprisingly, there was cross-reactivity between B177 and R465, and it involved a subset of T cells shared by the two epitopes (Figure 2) (30). This cross-reactivity was further validated by tolerance induction to R465, which compromised B177-induced protection against AA (30). This is a fine example of fortuitous mimicry between a pathogenic Bhsp65 epitope and a regulatory Rhsp65 epitope, and it might represent an inbuilt mechanism of a crosstalk between such pairs of epitopes to mediate regression of inflammation during the course of autoimmunity. In retrospect, a similar mechanism might offer additional explanation to the observations that a T cell clone (A2c) recognizing B180 was protective against AA (10) and that soluble B180 administered intraperitoneally (i.p.) prior to arthritis induction can suppress the development of AA (31).

## Antibodies to Hsp65 are Protective Rather than Pathogenic in AA

Adjuvant arthritis is believed to be a T cell-mediated disease. Unlike T cells, antibodies of arthritic rats fail to induce AA in naive recipients. Instead, anti-Hsp65 antibodies can mediate protection against AA. It was first shown that AA-resistant rat strains possess antibodies that are protective against AA, whereas susceptible rat strains, such as Lewis, lack these antibodies (32). Accordingly, the transfer of antibodies of resistant strain into susceptible strain induced protection against AA. Subsequent studies by others (8) and us (33) showed that such protective antibodies also develop in arthritic rats after recovery from AA. During the course of AA, eventually the antibody response became focused on few defined epitopes of Bhsp65 and Rhsp65 (8, 33). Furthermore, immunization of naive rats with specific peptide epitopes or anti-peptide antibodies, or sera from recovered rats, leads to protection against AA (Figure 1) (8, 33). The precise mechanism of protection is not yet fully defined, but one proposition is based on antibody-induced IL-10 production by PBMC (8). In a recent study (34), a humanized monoclonal antibody against Hsp60 (Prozumab) was reported to display disease-suppressive activity against AA and CIA. This antibody also can induce IL-10 in PBMC. Thus, antibodies against Hsp65 in AA are disease-regulating in nature.

## Responses to C-Terminal Determinants of Hsp65 are Involved in Environmental Modulation of AA

In a study on Fisher (F344) rats, whose susceptibility to arthritis is markedly modified by their housing environment, we showed that F344 rats raised in a barrier facility (BF-F344) are susceptible, whereas those raised in a conventional facility (CV-F344) were relatively resistant to AA (29). Similarly, F344 rats transferred from a barrier facility into a conventional facility (BF-CV-F344) acquired resistance to AA in a few weeks. In simple terms, BF and CV are also referred to as “clean” and “dirty” housing facilities, with the latter providing more opportunities for animals to be exposed to environmental microbes. We reasoned that Hsp65 of the environmental microbes might induce T cell response in CV-F344 that is cross-reactive with Bhsp65, and that these T cells in turn afford protection against AA. A similar situation might be expected for BF-F344 transferred into CV, whereas BF-344 will be spared as they are kept in strict controlled conditions. To test this idea, we examined the level of T cell response to BCTD and other peptides of Bhsp65 in these rats without any immunization with Mtb or Bhsp65 (29). Interestingly, CV-F344 spontaneously developed much higher level of T cell response to BCTD compared to BF-F344, whereas BF-CV-F344 were in between the two groups. Furthermore, adoptive transfer of spleen cells of CV-F344 rats, but not BF-F344 rats, reduced the severity of AA in recipients. Thus, BCTD-reactive T cells contribute not only to spontaneous regression of inflammation in Lewis rats (3) but also to environmental modulation of AA in F344 rats (29). However, the role of environmental factors on gut flora and its impact on AA remains to be defined.

## Use of Altered Peptide Ligands of Self-Hsp60 to Control Arthritic Inflammation

We have described above the use of native Hsp65 and its peptide epitopes in inhibition of arthritis. APLs, with specific amino acid residues of the original peptide modified to affect its binding to the major histocompatibility complex or the T cell receptor leading to altered T cell response, have also been explored for arthritis therapy (Figure 1). Initial work involved an APL of B180 containing alanine 183, which was shown to inhibit AA (35). It involved the generation of regulatory T cells and production of anti-inflammatory/immunomodulatory cytokines IL-4, IL-10, and TGF-β. Subsequently, APLs of human Hsp60 (e.g., APL-2 and APL-1) have been shown to suppress AA/CIA (36, 37). In addition, APL2 increased IL-10 production, but reduced IL-17 production by PBMC of RA patients (36), and it induced IL-10 production in PBMC of JIA patients (37). APL-1 was reported to induce Foxp3^+^ Treg coupled with apoptosis in activated CD4^+^ T cells in PBMC of active RA patients (38).

## Concluding Remarks

As elaborated above, studies on immune response to Hsp65 have offered several novel insights into both the induction and regulation of autoimmunity (3–9). Studies in the AA model have revealed Bhsp65 as the target of arthritogenic T cells. However, Bhsp65-reactive T cells also can be disease-regulating in nature, and such T cells demonstrate cross-reactivity with self-Hsp65 (3, 26). In addition, the T cells induced by self-Hsp65 also possess immunoregulatory activity against AA (11, 13) and dimethyl dioctadecyl ammonium bromide-induced arthritis (DIA) in Lewis rats (39). The regulatory role of self-Hsp65 is evident not only in the AA and DIA models but also in patients with JIA (22). On the contrary, in other disease conditions, such as diabetes (40) and atherosclerosis (41), pathogenic immune response has been shown to be directed against self-Hsp65. Nevertheless, Hsp65, its peptides, and APL can also be employed as immunomodulatory agents to attenuate these diseases. Thus, the dual role of Hsp65 has been reported not only in arthritis but also in diabetes, atherosclerosis, tumors, and transplantation (42–44). Further understanding of the cellular and molecular conditions that facilitate pathogenic versus regulatory immune responses to Hsp65 would pave the way for harnessing the immunomodulatory attributes of Hsp65 for therapeutic purposes in human diseases, as exemplified by the use of p277 of self-Hsp60 in human type 1 diabetes (45). This in turn would add novel agents to the therapeutic arsenal to control a variety of immune-mediated diseases.

In brief, the major new conceptual developments emerging from studies on immunity to Hsp65 in AA can be summarized as follows: (a) foreign–self antigen cross-reactivity, also referred to as “molecular mimicry,” has generally been viewed as a mechanism of induction of autoimmunity; however, studies in AA have shown that such a cross-reactivity (e.g., B256 and BCTD) can be immunoregulatory in nature; (b) most times, foreign–self cross-reactivity is limited to the corresponding homologous regions of the two proteins; in AA, a pathogenic epitope (B180/B177) can recruit a subset of T cells potentially reactive against a protective self-epitope (R465), thus elaborating a novel aspect of such cross-reactivity; (c) immune response to a self-antigen, which signifies a break in self-tolerance, has been the cornerstone of mechanisms to explain the initiation of autoimmunity; studies in AA have revealed that deliberate priming of self-reactive T cells using self-Hsp65 (human Hsp60 or rat Hsp65) as the immunogen, can effectively suppress arthritis; furthermore, the spontaneous emergence of anti-self T cell response during the course of AA can contribute to natural regression of autoimmune inflammation; (d) antibodies to foreign/self antigens are known to serve as mediators of immune pathology in a variety of autoimmune diseases; in AA, antibodies to Hsp65 have been shown to be protective against arthritis; and (e) previous work on peptide-based therapy of AA involved APL of peptides of foreign Hsp65 (Bhsp65); recent studies highlight that APL of self-Hsp60 also can serve as potent immunomodulatory agents.

## Author Contributions

EK, MD, YM, HK, and KM participated in writing of this article; EK and KM edited the article; and EK, MD, and KM prepared the figures.

## Conflict of Interest Statement

The authors declare that the research was conducted in the absence of any commercial or financial relationships that could be construed as a potential conflict of interest.
